# Supplementary material for: Attractors are less stable than their basins: Canalization creates a coherence gap in gene regulatory networks
Source: bioRxiv. 2025 Nov 8:2025.11.06.687062. Preprint. [Version 1] doi: 10.1101/2025.11.06.687062 (PMC12637702; doi:10.1101/2025.11.06.687062)
Supplement: 1 [file NIHPP2025.11.06.687062V1-supplement-1.pdf]

bioRxiv preprint doi: https://doi.org/10.1101/2025.11.06.687062; this version posted November 8, 2025. The copyright holder for this preprint (which was not certified by peer review) is the author/funder, who has granted bioRxiv a license to display the preprint in perpetuity. It is made available under aCC-BY 4.0 International license.

Supplementary Materials for

Attractors are less stable than their basins: Canalization creates a coherence gap in gene regulatory networks.

Venkata Sai Narayana Bavisetty, Matthew Wheeler, Claus Kadelka

Corresponding author: Claus Kadelka, ckadelka@iastate.edu

This PDF file includes:
Figs. S1 to S5

bioRxiv preprint doi: https://doi.org/10.1101/2025.11.06.687062; this version posted November 8, 2025. The copyright holder for this preprint (which was not certified by peer review) is the author/funder, who has granted bioRxiv a license to display the preprint in perpetuity. It is made available under aCC-BY 4.0 International license.

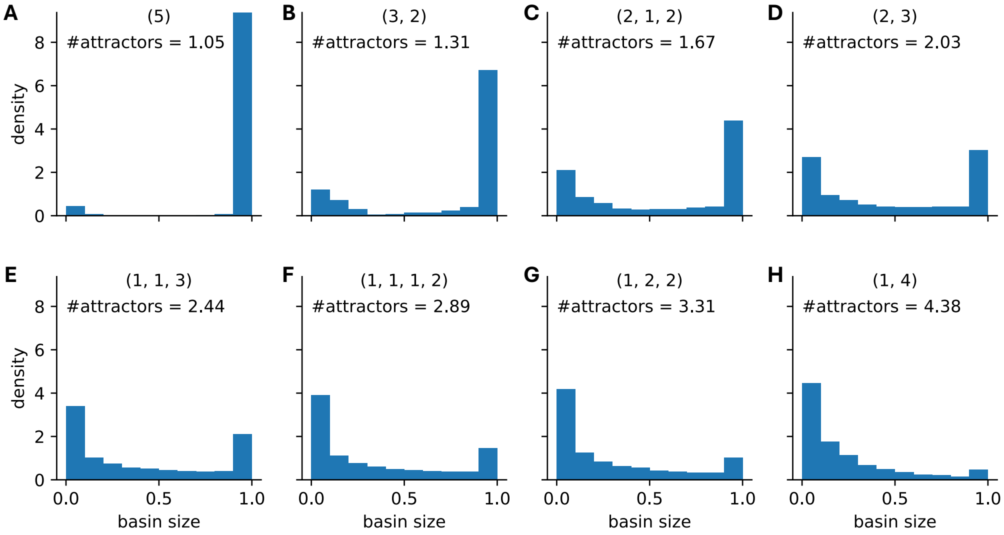

Figure S1: Basin size distribution for different types of nested canalizing Boolean networks. For 10,000 random 12-node BNs governed by 5-input nested canalizing update rules of different layer structure (specified above each panel), the distribution of the basin size is shown, in addition to the average number of network attractors.

bioRxiv preprint doi: https://doi.org/10.1101/2025.11.06.687062; this version posted November 8, 2025. The copyright holder for this preprint (which was not certified by peer review) is the author/funder, who has granted bioRxiv a license to display the preprint in perpetuity. It is made available under aCC-BY 4.0 International license.

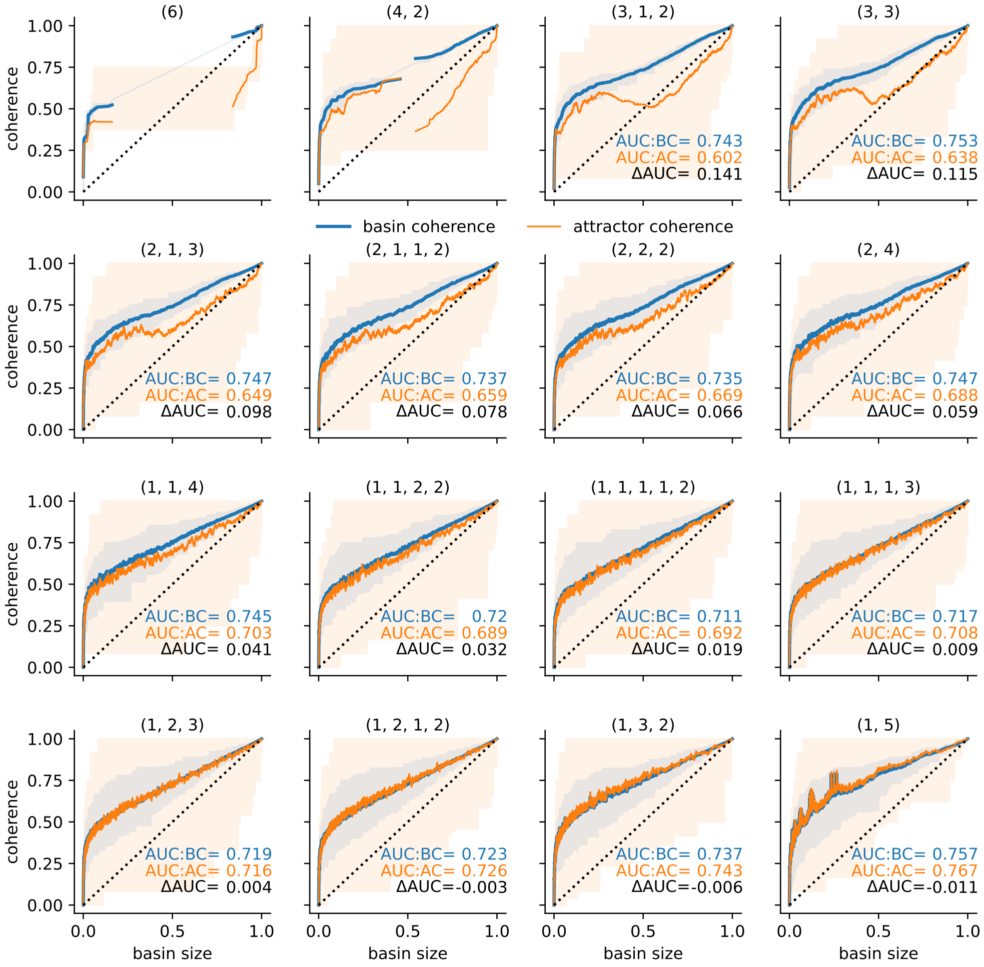

Figure S2: Robustness of nested canalizing Boolean network attractors and their basins. For 10,000 random 12-node BNs governed by 5-input nested canalizing update rules of different layer structure (specified above each panel), the coherence of each attractor (orange) and the coherence of its corresponding basin (blue) are stratified by basin size (x-axis). Shaded areas indicate the range of observed values. Lines depict rolling-window means with window size 50 and AUC is the area under the respective lines. The basin size distribution corresponding to each type of network is shown in Fig. S3.

bioRxiv preprint doi: https://doi.org/10.1101/2025.11.06.687062; this version posted November 8, 2025. The copyright holder for this preprint (which was not certified by peer review) is the author/funder, who has granted bioRxiv a license to display the preprint in perpetuity. It is made available under aCC-BY 4.0 International license.

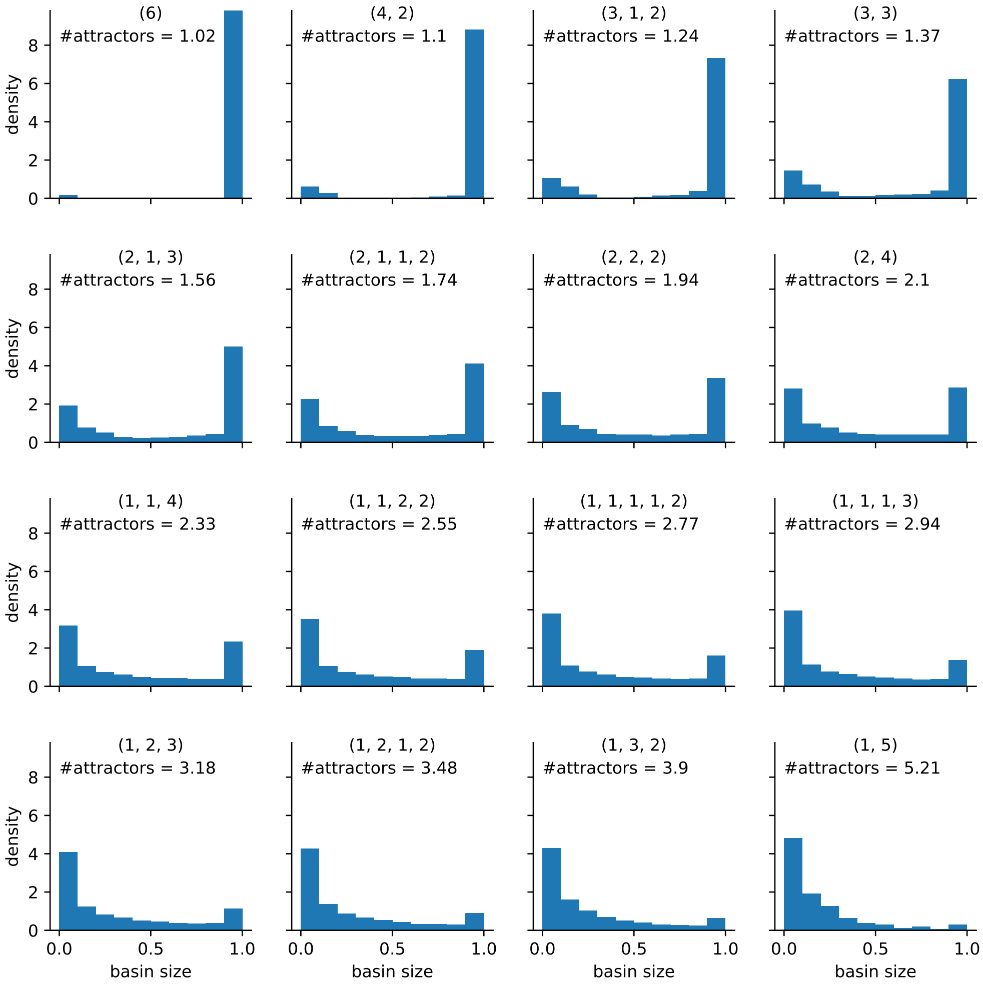

Figure S3: Basin size distribution for different types of nested canalizing Boolean networks. For 10,000 random 12-node BNs governed by 6-input nested canalizing update rules of different layer structure (specified above each panel), the distribution of the basin size is shown, in addition to the average number of network attractors.

bioRxiv preprint doi: https://doi.org/10.1101/2025.11.06.687062; this version posted November 8, 2025. The copyright holder for this preprint (which was not certified by peer review) is the author/funder, who has granted bioRxiv a license to display the preprint in perpetuity. It is made available under aCC-BY 4.0 International license.

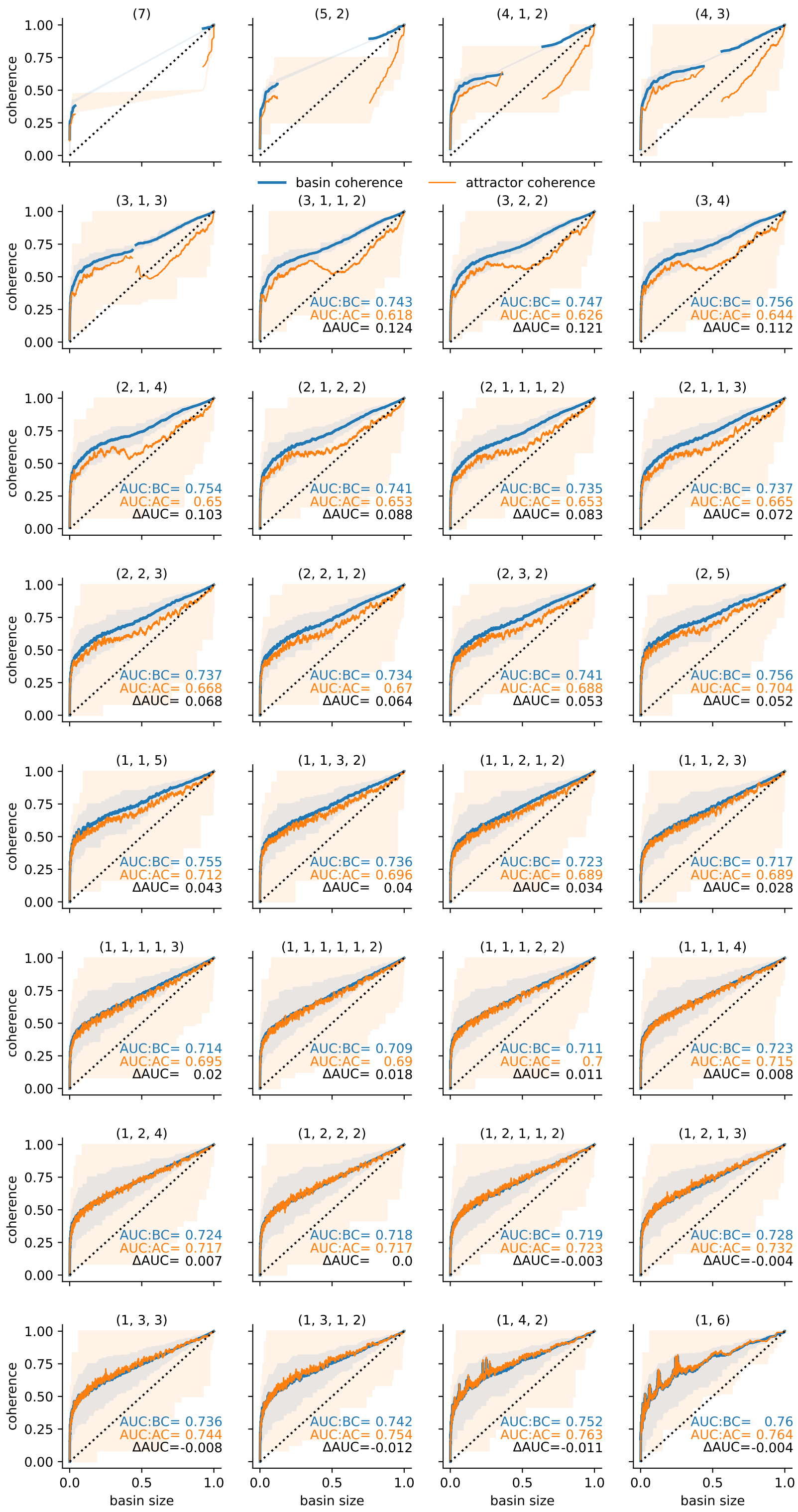

Figure S4: Robustness of nested canalizing Boolean network attractors and their basins. For 10,000 random 12-node BNs governed by 5-input nested canalizing update rules of different layer structure (specified above each panel), the coherence of each attractor (orange) and the coherence of its corresponding basin (blue) are stratified by basin size (x-axis). Shaded areas indicate the range of observed values. Lines depict rolling-window means with window size 50 and AUC is the area under the respective lines. The basin size distribution corresponding to each type of network is shown in Fig. S5.

bioRxiv preprint doi: https://doi.org/10.1101/2025.11.06.687062; this version posted November 8, 2025. The copyright holder for this preprint (which was not certified by peer review) is the author/funder, who has granted bioRxiv a license to display the preprint in perpetuity. It is made available under aCC-BY 4.0 International license.

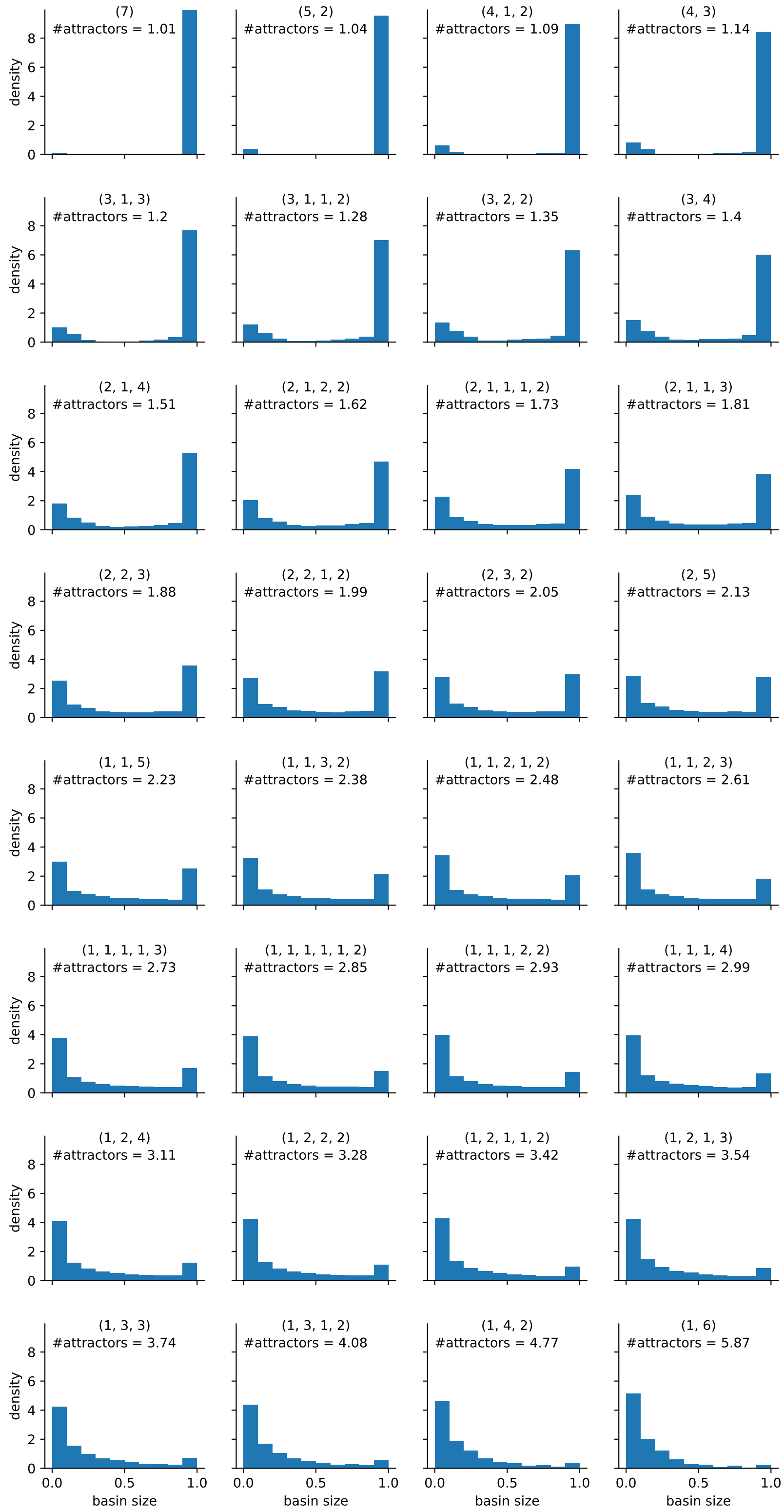

Figure S5: Basin size distribution for different types of nested canalizing Boolean networks. For 10,000 random 12-node BNs governed by 6-input nested canalizing update rules of different layer structure (specified above each panel), the distribution of the basin size is shown, in addition to the average number of network attractors.
